# Ten simple rules for getting the most out of a summer laboratory internship

**DOI:** 10.1371/journal.pcbi.1005606

**Published:** 2017-08-17

**Authors:** Toby P. Aicher, Dániel L. Barabási, Benjamin D. Harris, Ajay Nadig, Kaitlin L. Williams

**Affiliations:** 1 Department of Molecular Biology & Biochemistry, Middlebury College, Middlebury, Vermont, United States of America; 2 Department of Physics, University of Notre Dame, Notre Dame, Indiana, United States of America; 3 Department of Biology, Colgate University, Hamilton, New York, United States of America; 4 Department of Psychology, Northwestern University, Evanston, Illinois, United States of America; 5 Department of Life Sciences, Carroll University, Waukesha, Wisconsin, United States of America; Whitehead Institute, UNITED STATES

## Overview

Potential future scientists often gain their first exposure to real research practice through summer laboratory internships. Although these brief laboratory experiences are a major component of many public and private training initiatives, few written guide materials specifically address summer internships and how to optimally benefit from them. With that in mind, we have drawn on our summer research experiences to propose tips on how to approach all aspects of a summer internship, including planning ahead, navigating professional relationships, and maximizing impact, among others. We hope to open a conversation on how to enhance student experience at a critical early career juncture, when talented students are deciding whether to pursue a career in scientific research.

## Introduction

Perhaps you’re working in a laboratory at your college or traveling to a new institution in a far flung location. In either case, a research internship can be an incredible stepping stone in your professional, intellectual, and personal development. Over the summer, you can learn cutting edge techniques, expand your network, and refine your interests as you look towards graduate school or the job market.

The process of obtaining a summer internship can be as long and subjective as applying for or choosing an undergraduate institution. Summer research programs are increasingly competitive—some such as Amgen programs have 1,000 to 2,000 applicants for just 2 dozen spots. The strategies and specifics of acquiring a summer research internship could be a “Ten simple rules” post of its own, so we point readers to articles and tools that have explored this topic previously (S1 Table). Keep in mind that you don't have to be at a prestigious institution to learn new techniques, gain exposure to a new research field, and connect with scientists and other scientists-in-training.

Guidelines have been presented in this article series on how to approach undergraduate research in general [1]. Building on these helpful rules, we observe that summer research internships present unique challenges due to their immersive and time-limited nature. That being said, these short-term positions present a unique set of challenges that may prevent interns from getting the most out of the summer months. For instance, research projects often operate on the timescale of years, leaving it unclear how to best spend an 8 to 10 week internship. With busy summer schedules and unclear expectations, interns may end up completing laboratory procedures without intellectually engaging in their project. To help interns and mentors navigate these and other related issues, we have compiled advice based on our collective 16 summer research experiences. We hope that these suggestions will help interns optimally learn from and contribute to their lab. More broadly, by sharing these tips, we hope more interns will experience the thrills of laboratory research that have led all of us to pursue careers in science.

### Rule 1: Plan ahead!

Any project that you work on as an intern is probably part of an effort that spans multiple years. To have a meaningful internship experience, you need to have a small slice of that project that is both significant and doable in a short amount of time. It is not easy or obvious for your mentors to design a summer project that achieves both of these goals.

Planning ahead with your mentor can help make sure the internship experience is productive and engaging. In general, mentors who take on undergraduate summer students want to create great projects, but busy schedules may hinder extensive planning. Postdocs and graduate students may learn that they are expected to advise a summer student with a week or two of notice, and must cobble together suitable projects.

As soon as you are accepted to a summer internship, reach out to your PI to express (1) your excitement at working in the lab, (2) that you are eager to begin discussing your project with your direct supervisor, and (3) ask for reading material to gain background for further correspondence. Repeat these sentiments prior to your arrival as well, as a reminder of your upcoming presence. As you work through preliminary readings, connect back with your supervisor with sections that appealed to or challenged you. If you are able to successfully communicate in this way, supervisors can make subtle decisions in experimental animal allocation, surgery timing, and reagent ordering to facilitate a great summer project.

### Rule 2: Own your project

Your internship, while exciting and maybe entirely novel for you, will be a blip on the timeline of your scientific career. To really gain competence, you have to put a considerable amount of time and effort into learning and mastering new skills. Take the extra 3 steps to really understand why you’re pipetting liquid A into liquid B, and not the other way around. Recognize that protocols have intrinsic meaning, and you should know those meanings. You have been selected for what is likely a competitive and prestigious internship. Don’t be a benchwork drone. What makes you more valuable than the thermocycler? What are you doing? Why are you doing it?

In addition, owning your project means asking for help when necessary. It is, however, easy to fall into the trap of seeking continuous and excessive oversight from your coworkers or mentor. You want to ask for assistance and goals, but not instruction for every small step included in your project. Asking once is okay. Asking twice simply because you didn’t bother to write down the answer is annoying. If you have conducted research at your home institution, you will know that laboratory skills are often learned via trial and error over a long period of time. Summer internships afford no such luxury; it is critically important to optimize your learning by working diligently and efficiently. In cases where you continue to struggle with a concept, such as when your project has shifted directions midway, consider asking for a physical source, such as a paper or textbook, with which you can come to grips with it on your own.

It seems simple, but many undergraduates believe that they can remember everything said to them within the first few critical days in the lab. Your lab will likely gift you a notebook on your arrival, before they begin to enlighten you with the inner workings of every aspect of a protein you could imagine. Bring your lab notebook and writing instrument everywhere, and write down everything, thus developing a record of crucial methods, results, and insightful observations.

### Rule 3: Be humble

As an intern, you have to learn new techniques, get used to a new lab, and start a project that likely won’t be finished by the time you leave. So, while you should strive to do the best research possible and generate data useful to the lab, you’re unlikely to make a breakthrough discovery or get your name on a publication. Get used to this idea. Your mentor and your PI took you on because they think training the next generation of scientists is important and want to help you become a better scientist.

Be courteous to your mentor, PI, and lab. They made a choice to hire you as an intern, so make it worth their while. Always clean up your workspace when you’re done, attend lab meetings, and arrive to lab when your mentor requests. Don’t do anything inconsiderate to other members of the lab. Give your mentor and PI a thank you card or symbolic gift when you leave. You have a short time to make a good impression, and while your relationship with your summer PI may not be as well-established as with your home institution’s PI, you can still form a positive, potentially long-enduring, connection.

Don’t be too hard on yourself if you feel your project is moving slowly, isn’t going anywhere, or isn’t yielding interesting results. You should shoot for doing impressive work that advances a project but don’t let that define your experience.

### Rule 4: Be a team player

Your internship project probably represents a small sliver of a much larger project. Understand your lab's mission and figure out how a summer intern can best contribute. This will change based on a number of parameters, including the stage of the project, the career stage of your supervisor, and how your lab utilizes the summer months.

Suppose your advising postdoc arrived in the laboratory only a few months before you. In this case, you should learn how to help set up equipment and build analysis pipelines that can be used long after you’re gone. Alternatively, assume that your supervisor is in their sixth year with one foot out the door. Think of new analyses, and ask if you can play around with old data or help conceptualize results as they are being written up. If your lab supervisors are travelling to conferences all summer in a revolving door fashion, learn how to maintain their experiments and analyses, and become a reliable lab member who can hold down the fort. By recognizing what the situation is in your project and targeting your effort, you can have a larger impact on your lab and become an invaluable part of your team.

### Rule 5: Be a good collaborator

A successful internship is heavily dependent on having a good relationship with your mentor. Your mentor is responsible for giving you a project, teaching you new techniques, showing you around the lab, and helping you with problems that arise in your project. It is important that you establish a productive stream of communication with your mentor. Tell them if you don’t like your project or if you want to change directions. Summer research projects tend to be more narrowly focused than long-term undergraduate research goals, and with good reason. If you think your project is vague and undefined, ask your mentor or PI if they can give you more concrete work with better defined goals.

The goal of a summer internship is not to be a lab technician or an extra pair of hands to assist your mentor. Act as a collaborator instead of as an assistant; work to develop the concepts and methods of the project, rather than robotically completing task lists provided to you. Once you have put in the legwork to understand your project, think critically about it: Can the experimental paradigm be modified to better answer the research question? Are there newer analysis strategies that can facilitate greater insight into your data? Respectfully engage your supervisor on these ideas. Although research design may seem complex and out of reach for an intern, you are probably closer to this level than you think, and summer projects provide a great opportunity to hone your inquiry skills in a low-stakes environment with a supervisor to guide you. In doing so, you will get a better feel for the full scope of independent research, leave a stronger impression on your lab, and get a better letter of recommendation.

### Rule 6: Meet your PI 90% of the way

In many labs, face time with the PI is a precious resource, and access for interns may be limited. This is especially the case in the summer months, which are frequently used for conference travel and writing. Realize that although this is the case, your PI took on a summer intern for a reason, and generally has the best intention of being an active mentor. However, this will not be presented to you on a silver platter, and you must seek it out.

Is your PI travelling for most of the summer? E-mail them, and ask if they have any availability in the next month for a lunch. Does your PI mostly run between meetings during the day, and work in their office only at night? Stick around the lab at night, and pop into their office with intelligent questions about your project or one of their papers. Does your PI generally only meet with postdocs? Ask your supervisor if you can sit in on a meeting to discuss issues related to the project you are working on. Strong connections with PIs for undergraduates in science are rarely a given; they are mostly earned by go-getting mentees who are willing to meet advisors 90% of the way.

### Rule 7: Get to know your whole lab

Coming into your internship you most likely have a defined image for the ongoing work of your lab based on your literature review, e-mails, interviews, and project description. Unless you find yourself in a particularly small or specialized group, this will only be part of the picture. Some of your newfound coworkers may be working on problems that seem unrelated to your project. Your lab will also have a diverse scientific background amassed from pulling researchers from various undergraduate and graduate institutions.

Learn a bit about everyone you work with, and get at least a cursory understanding of their projects. For a larger group this may prove complicated. Say your mentor is dedicated to microbiology, but another portion of the group is heavy on the analytical chemistry, and the rest are sealed off in the nanotech room. Wrapping your head around these topics at various stretches of academia will take a lot of time you don’t have.

Instead, consider why individuals with diverging interests would be all under one group leader. Does nanotech provide some fundamental advantage to the analytical chemistry researchers, who can then aid the microbiologist aiming to halt the spread of disease? There must be some rhyme or reason, and understanding interdisciplinary interactions, large and small, will help you know where to look for new techniques later in your career.

It may be difficult during a few short weeks to become familiar with other researchers in your lab, especially if it is a large lab or people are away for summer conferences and presentations. Take advantage of going to lab meeting or going to lab social events to meet people in your lab and form connections. These gathering are a chance to talk about shared scientific interests and to connect to people on a personal level, both of which can help establish future professional connections.

### Rule 8: Get a feel for your data

Quantitative skills have become increasingly important in both research and industry career paths. Regardless of whether or not your internship has a programming course, you can start learning computational skills on your own. The simplest and easiest place to start learning quantitative skills is data analysis and presentation. Communicating information in posters, papers, or presentations requires you to interpret and display your data. In many cases you could get away with using software like Excel. But, by delving into more flexible and sophisticated tools, not only will you be able to have a more scientifically stimulating experience, you will also develop a skill set that is highly portable and will serve you, even if you don’t continue in research. Instead of using Excel or SPSS statistical software to analyze and display your results, try learning a programming language such as R or Python. There are plenty of online tutorials and resources specifically targeted for data analysis, including the article “Ten simple rules for better figures” published in *PLOS Computational Biology* [2].

In addition to statistics and figures, you could explore the theoretical basis of your research with quantitative skills. Biological systems often have mathematical models that represent the system over time—neurons, cardiac cells, circadian rhythms, gene networks, and metabolic processes all can be represented using sets of differential equations. Learning how to implement your related model and create simulations in a programming language will not only build your understanding of your research topic, but also help discover new dynamics of the biological system. Computational projects are great side projects for a summer of research because you can pick one up and put it down whenever it is convenient since it does not cost extra laboratory resources. The computational skills you develop, regardless of how far you delve into them, will be useful for any career path you choose.

### Rule 9: Balance reading and doing

Understanding scientific literature is an integral part of the research process, and a summer research position provides an opportune time to strengthen your reading skills. Your summer will most likely begin with background articles from your mentor, and you can continue to explore implicated concepts in weekly in-lab journal clubs. Additionally, many programs have journal clubs specifically for interns. Join one and become an active member; discussing the contents of a paper is an excellent way to check your understanding. One author found that they digest the article best if they skim it on the computer, then print it out and annotate. With every paper, consider the experimental procedure, how the analyses was performed, and finally how these connect to the author’s conclusions. If still in doubt, seek other articles that provide advice on reviewing scientific literature, but also seek your mentor’s and peers’ advice on how they tackle their reading.

However, you’re only at your internship for 10 weeks, and reading literature takes time away from working on your project. Striking a balance will help you get the most out of your time. This all depends on your background and your project. You can get by with reading enough to grasp an understanding of your project. For some well-defined projects that may be only a couple of papers. For projects that require you to design or select methods that may be a lot more. If your summer experience is in the same field as your home institution research, focus more upon specifics of your project and lab. Many internship programs assign students to their labs and projects, so even if you preference something you have a lot of experience in, you could end up in a totally different field. If you are entering an entirely new territory—like a cancer researcher entering a plant laboratory—spend some additional time beefing up your general knowledge of the field.

At some point you will be put on the spot to get your opinion on someone else’s work in the lab or on a paper the whole lab read. “I don’t know” is always an acceptable answer, but it shouldn’t be your default.

### Rule 10: Have fun

If all the above points on this list pan out, you’re going to have a thrilling experience, complete with a satisfying project, a supportive group, and unique social interactions. Your workday may begin with breakfast with coworkers, continue with in-depth research and group meetings, which will leave you tempted to follow-up with journal reviews in the evening. Immersing yourself like this will be exhausting!

It’ll prove beneficial to force yourself to step away from science every so often. The authors fondly remember playing volleyball as a break, thereby getting the exercise your brain and body needs. Take weekends to explore around your workplace. If you’re in a new location, then you have a myriad of novel opportunities to uncover nearby, but even if you stayed at your home institution, this could be a chance to move outside the collegiate bubble. Your best inspiration may come from an art gallery or a movie; giving your conscious scientific focus a break will let your subconscious make groundbreaking connections.

Depending on your excitement and work ethic regarding your project, this may be uncomfortable, even difficult. If you’re feeling guilty about taking some time to yourself, consider this: you may be the only one not doing so. At the end of the day, your PI and mentors go home to their families or roommates. They have dinner, maybe watch a movie with their kids. On weekends, they go on hikes, to a cool new art gallery, or a performance downtown. You probably do the same at college: classes, research and other commitments may take up most of your week, but the time in between is filled in with relaxing on the couch with friends. Research is a marathon, not a sprint, so take a break to leave yourself the stamina to stay in the race!

## Conclusion

Through the “Ten simple rules” and corresponding narratives, we explored the difficulties and realizations we have experienced through our summer internships. By addressing these hurdles, we hope to provide a smoother experience for you, and help you accomplish more with bolstered confidence. As final takeaway, we should note that your internship doesn’t end with your last day. Your coworkers, from peers to mentor to PI, will be interested in your ongoing trajectory. Keep in touch about your accomplishments, and consider any and all connections you have made as your greatest career resources. Your mentors should be happy to point out ongoing opportunities in the field, and perhaps more open than others about what to avoid on your scientific journey. Cherish this support, and be sure to pay it forward with your own mentees as you advance in your career.

## Supporting information

S1 Table Online resources for finding a summer internship. (DOCX)
