# Supplementary material for: Ten simple rules for getting the most out of a summer laboratory internship
Source: PLoS Comput Biol. 2017 Aug 17;13(8):e1005606. doi: 10.1371/journal.pcbi.1005606 (PMC5560542; doi:10.1371/journal.pcbi.1005606)
Supplement: S1 Table — (DOCX) [file pcbi.1005606.s001.docx]

**Supporting Information**

| Description | Link |
| --- | --- |
| Biomedical Research Internships List from Fred Hutchinson | [http://www.fredhutch.org/content/dam/public/](http://www.fredhutch.org/content/dam/public/education/surp/internships2016.pdf) [education/surp/internships2016.pdf](http://www.fredhutch.org/content/dam/public/education/surp/internships2016.pdf) |
| Summer Research  Opportunities from Pathways to Science | [http://www.pathwaystoscience.org/programs.aspx?](http://www.pathwaystoscience.org/programs.aspx?descriptorhub=SummerResearch_Summer%20Research%20Opportunity)  [descriptorhub=SummerResearch_Summer%20Research%](http://www.pathwaystoscience.org/programs.aspx?descriptorhub=SummerResearch_Summer%20Research%20Opportunity) [20Opportunity](http://www.pathwaystoscience.org/programs.aspx?descriptorhub=SummerResearch_Summer%20Research%20Opportunity) |
| List of Summer Re- search Opportunities for Undergraduates | [http://astronomy.mnstate.edu/cabanela/research/](http://astronomy.mnstate.edu/cabanela/research/reulist.php) [reulist.php](http://astronomy.mnstate.edu/cabanela/research/reulist.php) |
| How to Find a Sum- mer Research Intern- ship in Biology | [https://parasiteecology.wordpress.com/2014/01/21/](https://parasiteecology.wordpress.com/2014/01/21/how-to-find-a-summer-research-internship-in-biology/) [how-to-find-a-summer-research-internship-in-biology/](https://parasiteecology.wordpress.com/2014/01/21/how-to-find-a-summer-research-internship-in-biology/) |
| So you want to Apply for an REU... Here’s How | [https://astrobites.org/2013/01/05/](https://astrobites.org/2013/01/05/so-you-want-to-apply-for-an-reu-heres-how/)  [so-you-want-to-apply-for-an-reu-heres-how/](https://astrobites.org/2013/01/05/so-you-want-to-apply-for-an-reu-heres-how/) |

**S1 Table.** Resources For Finding an Internship

[1/1](#_bookmark0)
